# Cryogenic sequenced layering for the 3D reconstruction of biological objects

**DOI:** 10.1038/s41598-020-68682-z

**Published:** 2020-07-17

**Authors:** Vladimir Nikolaevich Nikolenko, Alexey Anatolyevich Terpilovsky, Alexey Leonidovich Kuzmin, Regina Alekseevna Lukashkina, Alexey Evgenievich Strizhkov, Andrei Vladimirovich Suslov, Ekaterina Vladimirovna Kochurova, Liliya Vladimirovna Gavrushova, Mikhail Yegorovich Sinelnikov

**Affiliations:** 1 Laboratory of Virtual Biology, village Red Pakhra, Rodnikovaya Street, 2, Moscow, Russia 108828; 2 Department of Human Anatomy, Sechenov University, Mohovaya, 11/10, Moscow, Russia 125009; 3 Department of Orthopedic Dentistry, Sechenov University, Tubetskaya, 8, Moscow, Russia 119048; 4 Department of Anatomy, Lomonosov Moscow State University, GSP-1, Leninskie Gory, Moscow, Russia 119991; 5 Institute for Regenerative Medicine, Sechenov University, Tubetskaya, 8, Moscow, Russia 119048

**Keywords:** Biotechnology, Software, Biomedical engineering, Imaging techniques

## Abstract

Three-dimensional (3D) visualization is applied throughout many specialities, prompting an important breakthrough in accessibility and modeling of data. Experimental rendering and computerized reconstruction of objects has influenced many scientific achievements, facilitating one of the greatest advancements in medical education since the first illustrated anatomy book changed specialist training forever. Modern medicine relies on detailed, high quality virtual models for educational, experimental and clinical purposes. Almost all current virtual visualization methods rely on object slicing producing serial sections, which can then be digitalized or analyzed manually. The tendency to computerize serial sections roots from convenience, accessibility, decent visualization quality and automation capabilities. Drawbacks of serial section imaging is tissue damage occurring within each consequent sectioning. To utilize the important aspects of real-life object reconstruction, and maintain integrity of biological structures, we suggest a novel method of low-temperature layering of objects for digitization and computerized virtual reconstruction. Here we show the process of consequent imaging of each novel layer of a biological object, which provides a computer with high quality data for virtual reconstruction and creation of a multidimensional real-life model. Our method prevents tissue deformation and biodegradation due to specific methods used in preparation of the biological object. The resulting images can be applied in surgical training, medical education and numerous scientific fields for realistic reconstruction of biological objects.

## Introduction

Three-dimensional reconstruction of objects is a necessary technology in furthering scientific progress. In health sciences, 3D technology presents opportunity for improved surgical training, diagnostic modeling, cancer studies, and is applied in biochemistry, regenerative medicine, reconstructive surgery and other numerous fields^1–9^. Current methods of morphological analysis require fixation, embedding, sectioning, after which the acquired samples can be analyzed^10–15^. Sectioning is most commonly performed via microtome slicing, producing 5–20 μm thick slices. This method of microtome slicing includes mechanical separation of fixed and embedded biological tissues and prior chemical exposition, which often leads to damage and disfigurement of biological material. The preservation of biological tissue integrity and achievement of quality computerized visualization is necessary for future use of acquired images in studies and experimental modeling, which have a variety of applications.

Technology of three-dimensional reconstruction of biological objects has been in development for the past 25–30 years. Premier projects with multidimensional reconstruction included the Visible Human and the VOXEL-MAN projects. Visible Human developed a digitized sequence of 1871 cross-sections of a male human body with a step of 1 mm, and 5,000 cross-sections of a female human body (0.33 mm step)^16^. VOXEL-MAN used data from the Visible Human project to develop a 3D educational software^17^. An article published by authors Sieber et al. discusses a method of temporal bone modeling using a method of micro-slicing with digitization. The authors claim several procedural inaccuracies due to chemical dehydration, geometric deformations due to sequential grinding and micro-slicing, stitching instability^18^. In order to exclude possible inaccuracies, cryogenic preparation and high quality milling should be carried out. A complete and more accurate rendering can be achieved by consequent removal of layers from a specimen with digitization of each novel surface.

**Figure 1:**
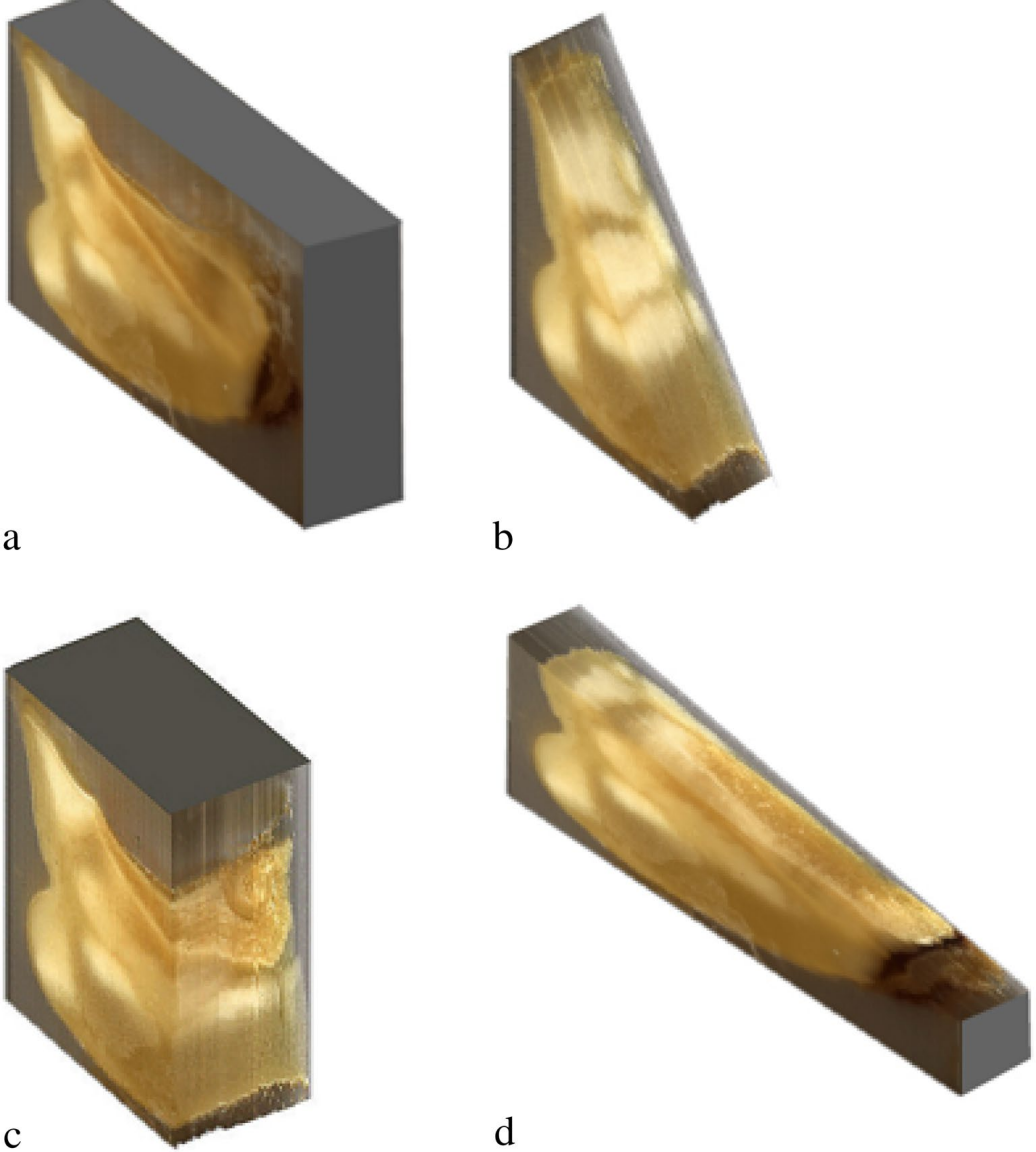
Layered reconstruction of a sectioned corn pellet. Visualized embedded in paraffin [(**a**) axonometric reconstruction; (**b**) diagonal cut of digitized pellet; (**c**) transverse cut of digitized pellet; (**d**) longitudinal cut of digitized pellet].

**Figure 2:**
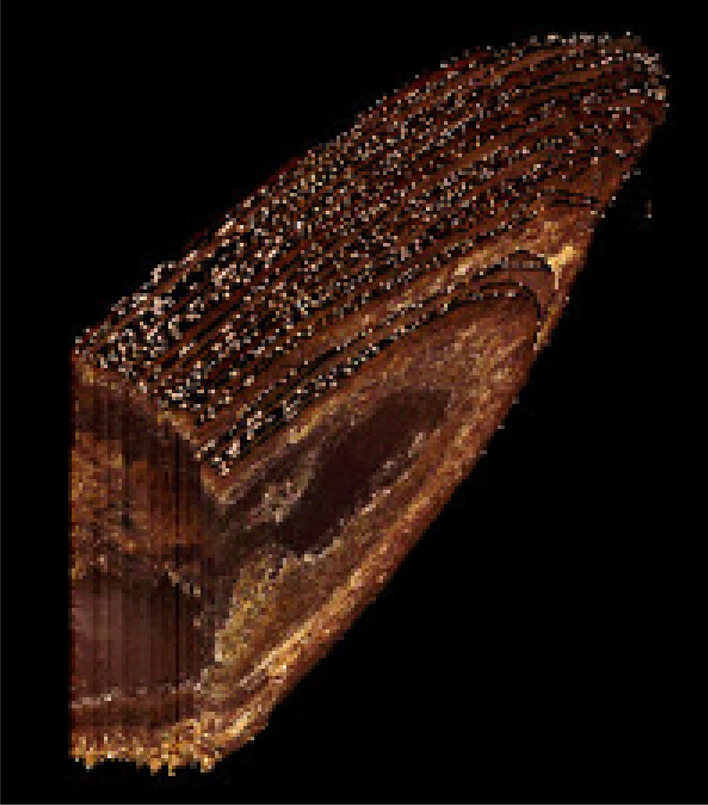
Layered reconstruction of a sectioned spruce seed. Three-dimensional reconstruction with removed molding material. Layering step 10 μm.

**Figure 3:**
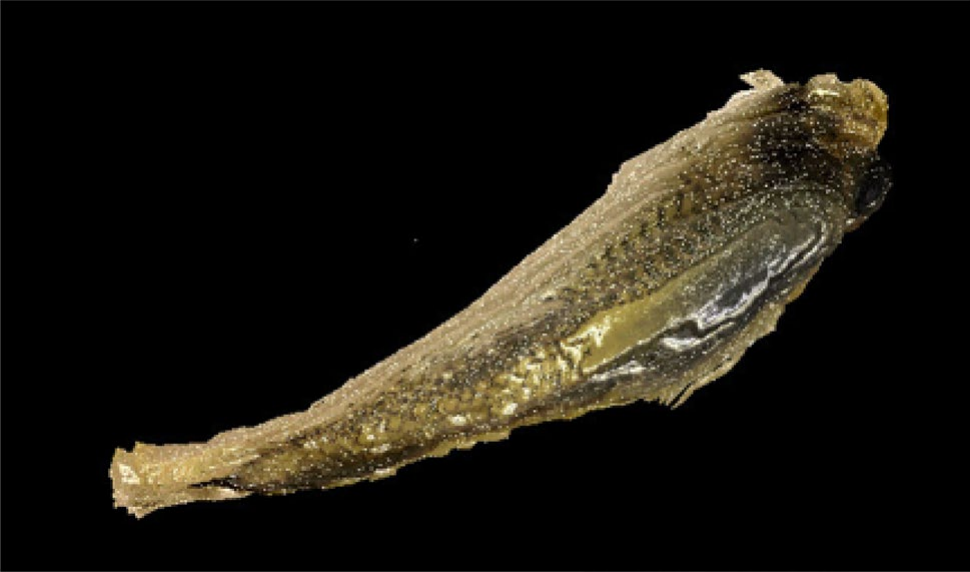
Layered reconstruction of sectioned rainbow fish. Paraffin removed. 10 μm step.

Our personal experience with layered reconstruction using series of sections was carried out on small biological samples, including a corn pellet, spruce seed and a rainbow fish. The corn pellet can be seen in primary multidimensional reconstruction with moulding material still in place (Fig. 1). Computerized removal of molding material provides better visualization and multidimensional modeling (Figs. 2, 3). Our attempts with virtual 3D reconstruction of microtome sectioned material showed significant drawbacks of this method, due to notable tissue deformation and abundance of artifacts. This was due to the fact that each layer was deformed during preparation of sections, making impossible proper image layering. Consequentially, we began development of a method that would account for these deficiencies.

Modern biological object imaging in different specialties requires precise, high-quality and multidimensional capabilities. Special interest to layered object visualization is regarded in medical pathology. Histological and microscopic imaging highly depends on mechanical sectioning, which is often performed with a significant interval between layers to minimize tissue deformation. Three-dimensional visualization with a low interval between layers and minimal tissue deformation is required for correction of technical inaccuracies. Layered, multidimensional, computerized imaging with minimal structural damage is the next step in object visualization technology, which can facilitate a new age of scientific discovery.

## Results

We developed a novel method of virtual reconstruction of biological objects by sequenced layering via high-precision low temperature micro-milling of flash-frozen embedded specimen and consequent digitization. Flash-freezing of biological material allows for the elimination of the need for chemical preparation, which is performed in classical methods of fixation for tissue dehydration, and is known to cause structural damage. The proposed method accounts for different physical and chemical attributes of biological objects, preserving the entire specimen in exact conditions upon cryogenic conservation. Digitization and computerized rendering with multidimensional modeling are performed using successive surface layer images. After complete micro-milling of entire cryogenic specimen, a three-dimensional model consisting of discrete high quality layered elements is attained using captured surface images from each novel layer. The layer step is minimal due to specific cryogenic micro-milling technology allowing for a step of as little as 2 μm.

**Figure 4:**
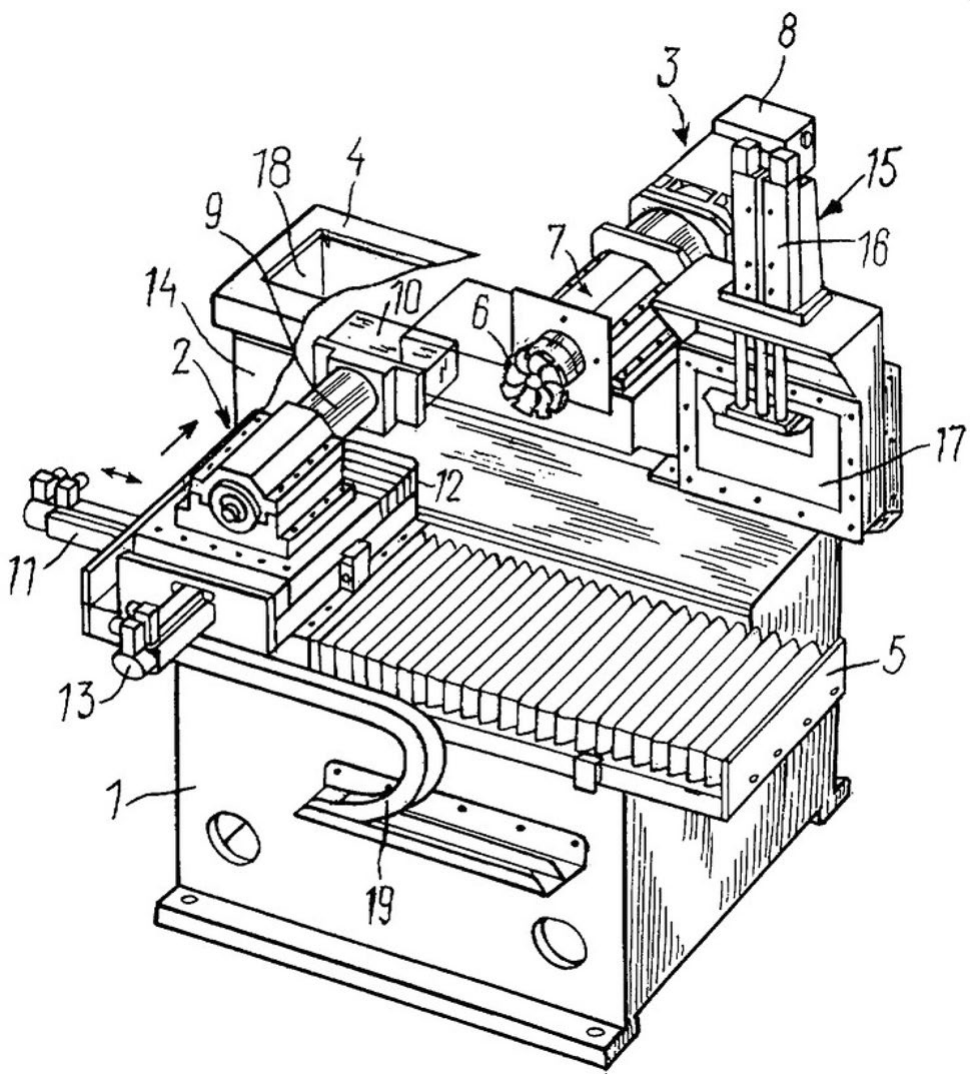
General view of device for virtual reconstruction of biological objects; anterior view (1—anti-vibration base; 2—object fixation device; 3—face micro-milling structure; 4—cryogenic chamber for flash-frozen object; 5—primary table; 6—face micro-mill; 7—spindle mount; 8—electric motor (outside cryogenic chamber); 9—stability pallet; 10—frozen and embedded biological object; 11—longitudinal linear movement mechanism; 12—secondary table mount; 13—transverse linear movement mechanism; 14—anterior cryogenic chamber wall; 15—digitization window management structure; 16—window transition unit; 17—damper; 18—cryogenic chamber cover; 19—flexible minimally resistant cable.

**Figure 5:**
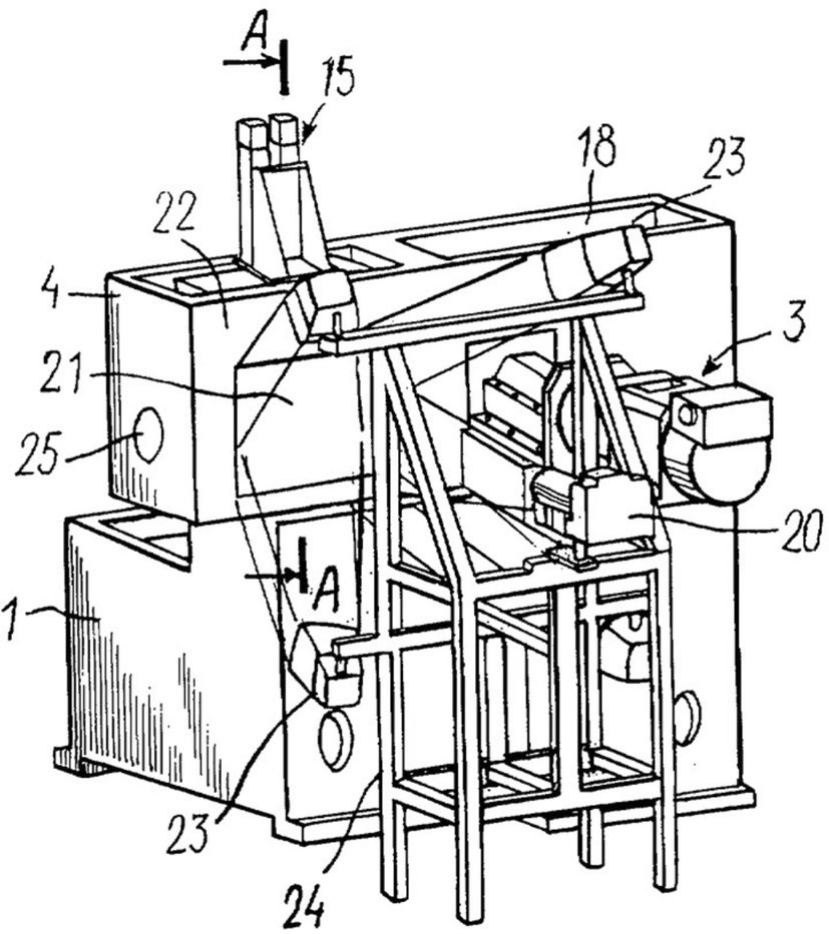
Posterior view of device for virtual reconstruction of biological objects (1—anti-vibration base; 3—face micro-milling structure; 4—cryogenic chamber for flash-frozen object; 15—digitization window management structure; 18—cryogenic chamber cover; 20—digital camera; 21—digitization window; 22—posterior cryogenic chamber wall; 23—lighting fixtures; 24—supporting structure for digitization device and lighting sources; 25—compressor (not shown) access to cryogenic chamber; A–A—digitization window closure line.

A low-temperature face micro-milling construction was built for this project, the structure of the negative-temperature milling device includes a cryogenic chamber for the fixation of the biological object, a face micro-milling installment for repeated interaction with the specimen, a digitization device and lighting sources (Fig. 4, 5).

The result of the developed virtual reconstruction device is a series of discrete digital elements (layers), acquired from the surface of a flash-frozen embedded biological object in the cryogenic chamber with step-by-step consecutive micro-milling and digitization of novel layers. The superior feature of this device is elimination of object slicing, therefore the characteristic integrity of the biological tissues is maintained, preventing tissue deformation of different sorts. The process of flash-freezing of the biological object with consequent low-temperature embedding and secondary flash-freezing of the embedded biological object provides overall tissue stability during precision layered micro-milling in negative-temperatures.

The developed method for virtual reconstruction of biological objects was applied in several trial cases. In each case, the biological object was flash-frozen using liquid nitrogen at − 196 °C in the cryogenic chamber. No additional chemical preparation of the biological object is required prior to freezing. No staining is required. Upon freezing, the biological object was embedded with a cryogenic medium—*Richard-Allan* *Scientific* *Neg-50* *Frozen* *Section* *Medium* was used. The embedded object was then frozen along with the medium using rapid liquid nitrogen freezing. The dual freezing algorithm was used for retention of native tissue geometry and color characteristics throughout preparation. The formed frozen complex was maintained at a temperature below − 30 °C and humidity of 25–30% in the cryogenic chamber. These parameters allow for tissue integrity stability and proper layering and digitization quality.

The developed technique allows for individualized adjustment of parameters, such as freezing temperature, method of embedding, cryogenic chamber environment, milling rate and layering step, as well as digitization modification. The layering step plays an important role in the final 3D rendering. A lower step rate accounts for a lesser rate of inconsistency between layers, and minimizes the occurrence of deformations needing adjustment. The camera used for large-scale digital rendering can be exchanged for a microphotography camera for microscopic visualization. A camera upgrade does not require any change to the digitization process, as both the cryogenic milling device and the computer rendering software independently function irregardless of image capturing appliance. Devitalization prior to flash-freezing can be performed with formaldehyde vapors if necessary.

**Figure 6:**
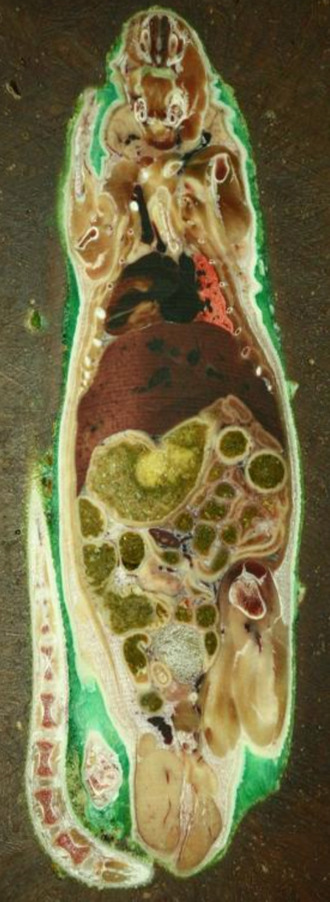
Transverse sections of laboratory rat, frozen, fixed, embedded, milled.

**Figure 7:**
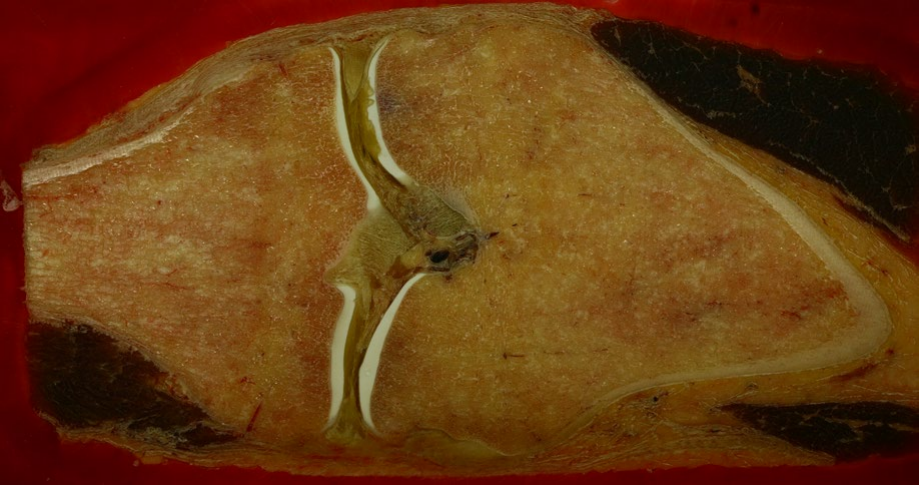
Cross section of a knee joint, digitization image for computer rendering. Normal anatomical structures are intact, no deformation is seen.

**Figure 8:**
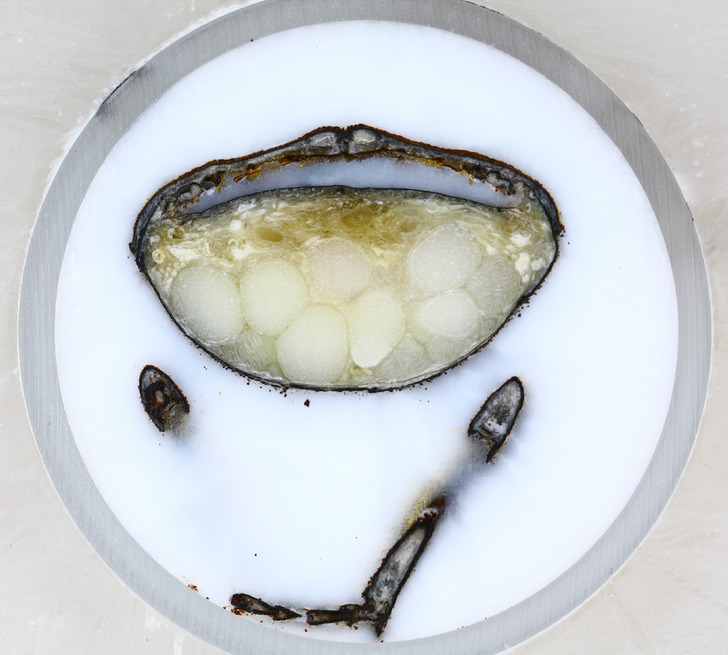
Beetle surface image after micro-milling. Notice intact eggs and chitin structure.

High quality imaging with a 5–20 μm step rate was tested on laboratory rats. As a result a detailed rendering was reconstructed. The quality of the surface images were exceptional, the surface of the milled and polished material proved to be without notable deficiencies (Fig. 6). High quality digitization of human tissue was also performed (Fig. 7), in both cases the surface texture of the embedded object was smooth and no tissue deformation was noted. An experimental three-dimensional reconstruction of a beetle surprised us with present eggs, which were completely preserved throughout virtual reconstruction. Chitin did not require staining for proper visualization (Fig. 8).

**Figure 9:**
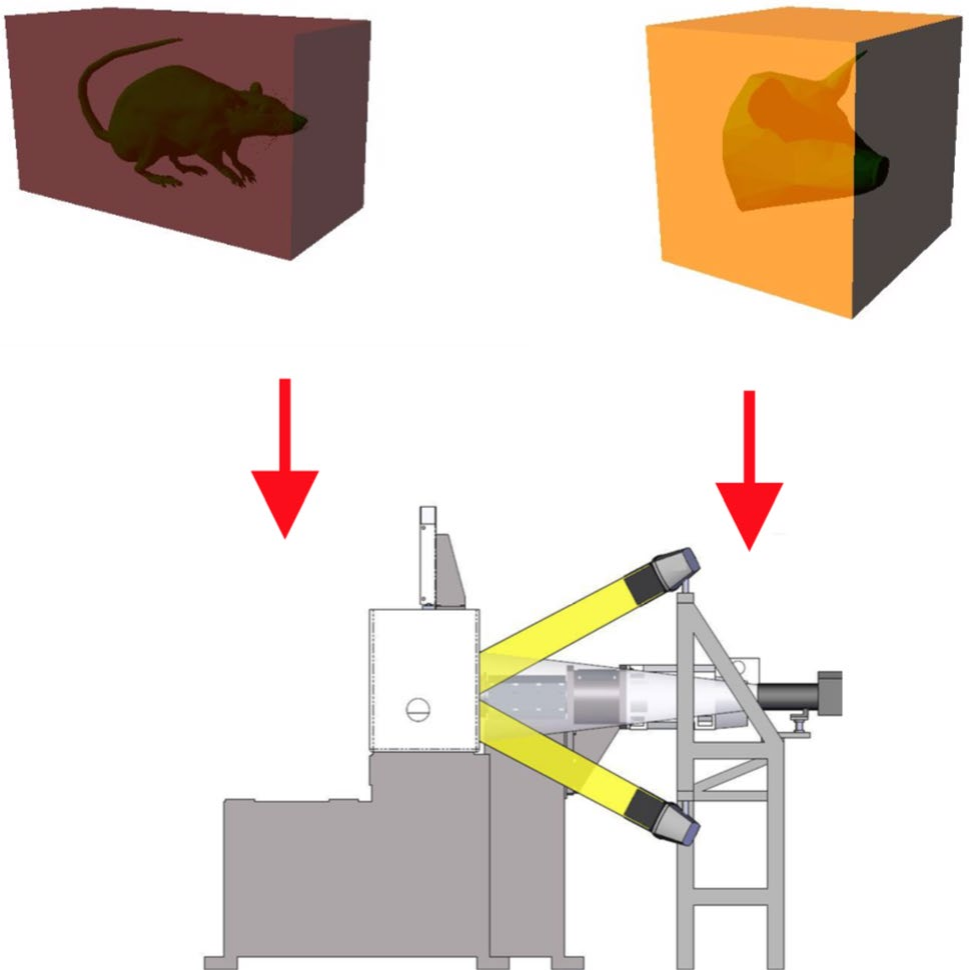
Schematic interpretation of digitization process. Frozen embedded biological objects are placed into the cryogenic chamber of the micro-milling device, where they undergo layered digitization.

Computerized rendering of the acquired images was performed by layered sequencing and digital model reconstruction. Modifiable parameters such as lighting, image resolution, zoom and quality, milling step and color parameters—all influence the process of model digitization. The process of virtual reconstruction commences with image capturing. A swine head and an adult rat were used as subjects for this process. Upon flash-freezing, embedding and secondary freezing, the models were placed in the cryogenic chamber of the negative temperature micro-milling device for digitization (Fig. 9).

**Figure 10:**
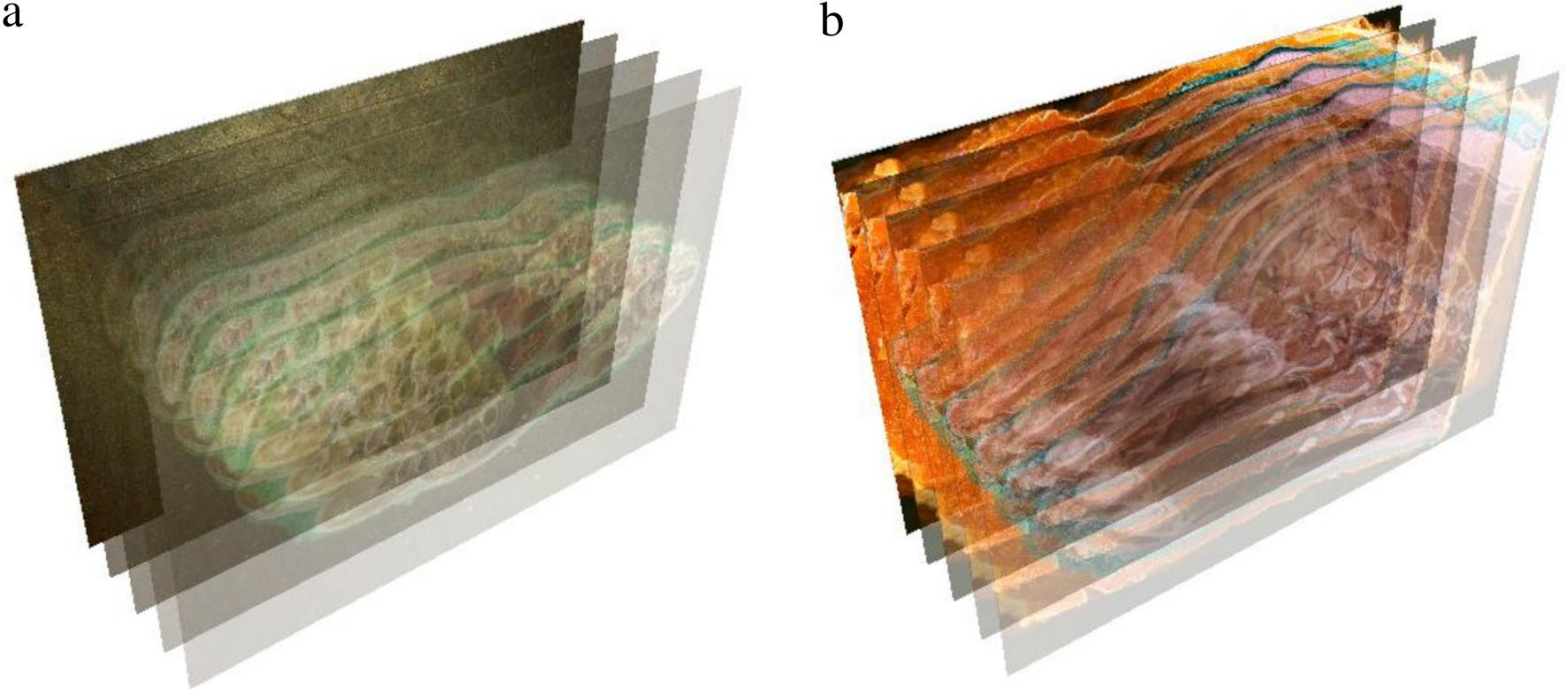
Model of layered image sequence [(**a**)—adult rat; (**b**) swine head] (Selena software v.8.0. https://vbiol.ru).

In the cryogenic chamber, at working temperature (maintained at and below − 30 °C) and humidity (25–30%), the experimental subjects underwent sequenced micro-milling. After each step, a photo of the model surface was captured. Therefore the polished via micro-milling surface is captured, the removed layer is discarded. This provides highest tissue integrity and morphological quality. As a result a series of images was captured (Fig. 10).

**Figure 11:**
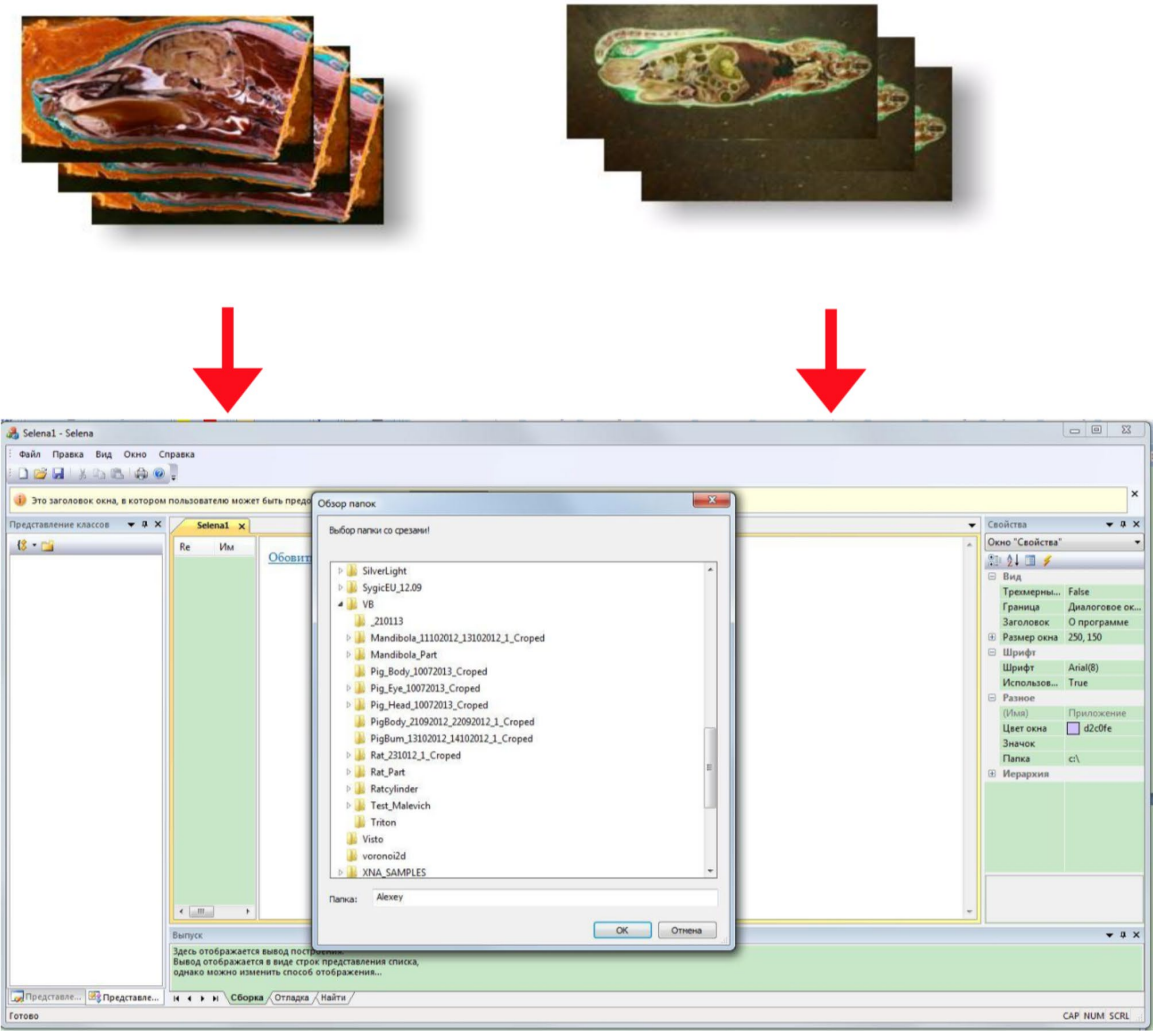
Selena software (v.8.0) for image processing and model rendering (https://vbiol.ru).

Overall 4,523 layers (each 4,368 × 2,912 pixels) were acquired from the adult rat (step of 10 μm), 5,358 layers (each 4,368 × 2,912 pixels) from the swine head (step of 20 μm). Computerized rendering is performed through Selena software, Version 8.0 (Fig. 11).

**Figure 12:**
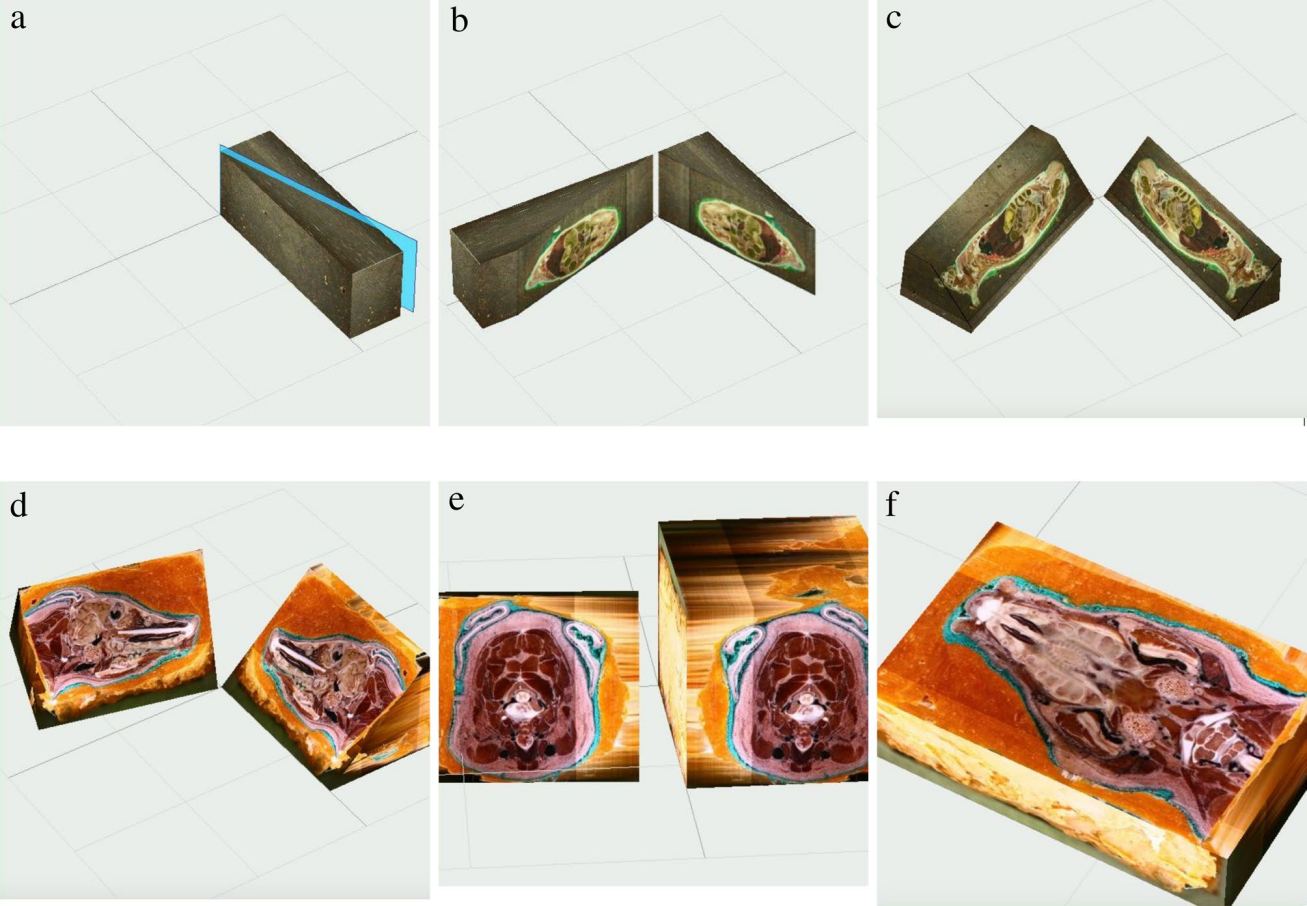
Model slicing in third party software (VBIO laboratory software (Selena software v.8.0. https://vbiol.ru) [(**a**–**c**) examples of adult rat model slicing; (**d**–**f**) examples of swine head model slicing].

The acquired models undergo required modification and can be forwarded for further production in more sophisticated software, such as TRIO or VBIO. Essentially, data analysis depends on researcher preference. As an example, an internet platform VBIO is developed with MODEX computing core for three-dimensional slicing of the model (Fig. 12).

Comparison to classical methods of histological staining through RGB-profiling, evaluation of optic density and surface profiling was performed. A total of 1,200–2,400 images (1,500 × 1,250 pixels) were generated in .tiff format for each of the 12 rat knee cartilages. Main morphometric results of both methods can be seen in Table 1.

**Table 1:** Morphometric comparison of rat knee cartilage features using two different methods of preperation.

Criterion	Classical histological slicing	Cryogenic sequenced layering	*p* value
Radial thickness of cartilage, mkm	185 ± 15	232 ± 15	0.032*
Cartilage surface factor	1.08 ± 0.8	1.16 ± 0.5	0.942

*Statistically significant findings.

Increased radial thickness of cartilage in the novel method is characteristic of decreased tissue deformation. The similarity in cartilage surface factor results indicates diagnostic integrity of the material in both methods.

The developed method of three-dimensional reconstruction of biological objects proved to be successful. Several dozen models were reconstructed using this method. In each case the images were analyzed for tissue deformation and integrity of biological structures by a pathologist. No significant deficiencies were found. Overall, this method of extracting data from the surface of a prepared cryogenic model provides greater morphological integrity than computerized layering of separate sections.

## Discussion

Evolution of three-dimensional technology has persistently influenced medical research. 3D printing, modeling of surgical procedures, training, diagnostic procedures, cancer studies all utilize the capabilities of multidimensional reproduction of biological tissues. Our method of virtual reconstruction of biological objects via computerized rendering of a series of surface images from a cryogenic model is dedicated to perfecting the technology of layering of digitized micro-slices. Instead of digitizing separate histological sections, we captured the surface of the model after each polish with a micro-mill. The resulting series of surface images are analyzed by special software and consequent object rendering and reconstruction is performed. Though we used Selena software for our models, the choice of software depends on researcher preference and availability.

Our method significantly differs from existing methods of optic sectioning microscopy and other tissue integrity preserving methods of 3D reconstruction of biological objects. This is mainly due to the methods of sample preparation and sectioning technology. Most classic methods of optic microscopy are based on detection of scattered light from the evaluated object. Scanning optic microscopy allows for multidimensional scanning, which provides the ability to reconstruct bitmap surface images of exceptionally high quality. As a result, three dimensional textures of sample surfaces can be visualized. Scanning probe microscopy specializes in acquiring surface images with regard to local characteristics, and as a result provide significant data on the topographic textural aspects of the examined sample. Our method specializes in acquiring layered information from a multitexture surface, the geometry of which is close to that of a plane. Therefore, the optic (bitmap) qualities of each separate layer on the surface of the frozen biological sample are captured and processed. Each layer of the frozen intact biological sample undergo high precision milling, uncovering an intact lower layer. The surface area for layered reconstruction is significantly larger than in all other described visualization methods. The resulting surface images makeup an archive of images, which can be layered and analyzed according to research specifications. The layered images of milled surfaces then undergo 3D rendering, providing an intricately complete virtual reconstruction of the biological sample in all and with the ability for computerized sectioning and histological evaluation.

Confocal laser scanning microscopy allows for visualization of cellular structure, yet the depth and area of optic manipulations is greatly limited by a number of parameters (including wave length and sample optic properties. Our methods improves on this by offering visualization of morphological and topographical surface properties not only on the cellular level, but within the range of the intact biological object per layer. Our method allows for application of fluorescent and radiological examination of samples, since the technology behind the method does not limit their applicability, as they can be applied prior to cryogenic processing. Apart from a significantly larger capability for tissue sectioning, which offers the ability to work with intact biological samples, our method can be applied in reconstruction of a high variety of materials, including titanium implants, medical instruments and implants, offering the ability to capture the state of interaction between tissue and foreign object.

In a study by Roy et al.^19^, the authors discuss a method of obtaining thin sections by a cryomicrotome, studied using light and fluorescence microscopy. The disadvantage of this method is the inevitable deformation of the material in the preparation of slices, which requires thawing of slices, moving them to a microscope for study and photographing, the high thickness of slices (5 mm) and inability to evaluate hard structural components (such as titanium implants. With our method, the study is carried out directly on the cryoblock manufactured at the beginning of the study, which located in the cryochamber throughout the study. With high-precision milling, a thin layer of material is removed from the front surface of the cryoblock, therfore(no cut is made. The raster image is fixed from the new cryoblock surface formed as a result of milling. This eliminates significant deformation of the material and significantly increases the accuracy of the study. The technological features of the cryogenic milling device allow for slicing through hardened materials integrated in the biological tissue, such as titanium implants, chitin, dental implants, metals.

Parrif G. J. conducted a study on the working surface of a cryoblock with dimensions of 30 × 30 μm^20^. Most of the anatomical structures of animals and humans are much larger, therefore we created a working cryochamber surface of 100 × 100 × 100 mm, increasing the surface of each layer by over 10^7^. Olivier Salvado et al. developed a method of 3D cryo-section imaging of blood vesssels lesions for validation of MRI data. Their method involves classic histological sectioning of soft tissues for immunofluorescent microscopy. The main drawback of this method is size of layered sections, the use of histological stains and fixators, and inability to slice high density materials, such as metals. Our method improves on these limitations by expanding the surface of each layer, decreasing the step between each surface image (to under 5 μm and developing a high-precision milling device, capable of slicing through high density materials, therefore significantly expanding the possibility for research application.

Overall this method improves on existing methods of virtual reconstruction of a series of images, but still several drawbacks are recognized. Maintenance of cryogenic temperatures throughout the procedure of virtual reconstruction requires high energy expenditure. Transportation of the model is limited due to the need to maintain a stable low temperature for purity of results. The developed low temperature face micro-milling device requires regular maintenance. The presence of mechanical disturbance (operator influence, external vibrations) can influence the precision of micro-milling, therefore the proposed method should be carried out in a protected environment. A temperature increase during milling can be seen and should be accounted for by lowering the temperature in the cryogenic chamber prior to milling. Minor inaccuracies due to accidental camera movement can cause stitching inaccuracy and should be accounted for. Computerized extraction of the embedding material requires perfection, the outline of specimens is not always properly identified, which can cause image artifacts. The cryogenic chamber parameters are currently 200 × 100 × 100 mm, limiting the size of the biological object, but the chamber size can be modified. These drawbacks represent a basis for further development and perfection of this novel method.

The proposed method can facilitate significant educational advances by creating highly accurate models of normal anatomy and pathology. Modeling of anatomical objects through virtual reconstruction and three-dimensional rendering can provide a valuable database of graphic material. More so, 3D models of anatomical regions will allow surgeons to improve their skills by working with realistic environments. The importance of surgical training on highly realistic digitized models is apparent^21^. The digital age can provide surgeons with valuable materials for educational, practical and experimental purposes.

**Figure 13:**
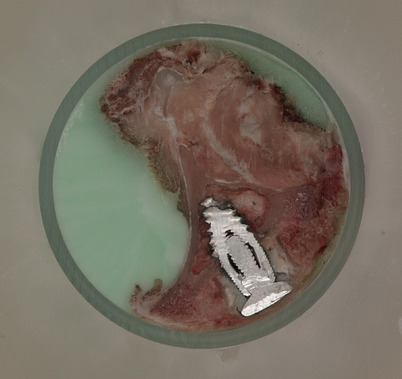
Knee joint of rabbit with titanium implant—surface image of milled cryogenic specimen.

The described method and device for virtual reconstruction of biological objects is highly modifiable and can be adjusted according to required researcher parameters. The milling nozzle can be interchanged depending on polishable material. We had experience with milling of a knee joint with a titanium implant present, no procedural complications were noted (Fig. 13).

The camera and lighting can be changed according to researcher choice. Microscopic capturing of histological material is possible by using microphotography capable cameras. Temperature control within the cryogenic chamber can be modified, but is recommended to be maintained below − 25 °C, due to the formation of intracellular ice at − 25 °C^22^. The distance between layers can be regulated by adjustment of device step parameters. The cryogenic chamber volume can be modified. The method for cryopreservation, freezing and embedding can be changed when required, but the algorithm of biological object preparation and virtual reconstruction remains the same.

Increased availability of high quality imaging and real life morphology rendering will provide a valuable source for educational, experimental and theoretical work. Our method of cryogenic biological object preparation for virtual 3D reconstruction can provide a substantial input towards the development of educational databases, surgical training models and virtual anatomical theaters.

## Conclusions

Throughout a 20 year history of development, the cryogenic preparation and working conditions for layered milling and virtual reconstruction using sequenced surface images has proven to be a successful method of tissue-preserving biological object reconstruction in our practice. A minimization of tissue damage was seen due to the characteristics of the preparation process. High modifiability of the proposed method provide vast applicability and effectiveness. This technology has influenced the commencement of numerous projects on virtual reconstruction of anatomical structures, modeling of surgical procedures and virtual pathology biobank production. Overall, flash-freezing of biological objects with consequent embedding and secondary freezing provide a highly stable model for sequenced layering and virtual reconstruction. The quality of the produced images is exceptional, though depends on method of visualization, which can be modified according to researcher preference. Consequent computerized modeling does not require specific software, and can be performed with any system of choice, capable of working with an input of stacked images.

## Materials and methods

All experimental protocols were carried out in accordance with relevant institutional and local guidelines and regulations and were approved by I. M. Sechenov First Moscow State Medical University licensing committee. *Selection* *of* *biological* *objects.* All biological objects were categorized into two groups. Group 1 included human tissues, group 2 consisted of animal tissues. The animal and human tissues used during the development of this method did not undergo any preparatory procedure prior to cryopreservation. All biological objects used in this study were acquired from a university vivarium and pathology department and were not used in any other experimental studies. The selection criteria for biological objects was not strictly defined, as the process for virtual reconstruction with cryogenic sequenced layering does not require special preparation. Generally, cryopreservation of biological objects was performed 2–48 h post-mortem. Biological objects with inclusions of non-organic materials were not excluded from the experiment, though milling corrections were required. Previously devitalized objects using formaldehyde vapors can be used.

To methodologically evaluate the advantages of this method, we performed a comparative morphometric analysis of our method of cryogenic sequenced layering and classic histological slicing. 12 rat knee cartilage reconstructions were prepared using the cryogenic sequenced layering method, and 12 rat knee cartilages were prepared using fixation in 10% formaldehyde (pH = 7.4), decalcification with “Cal-Ex” (Fisher Scientific), fixed in paraffin, staining with Hematoxylin & Eosyn and safranin O. Morphometric evaluation was carried out in accordance with the principles of quantitative image analysis using the *ImageJ* free access software (USA, https://imagej.net). The radial thickness of the cartilage (μm), the surface factor (ratio of the length of the curved segment corresponding to the actual border of the cartilage, between points at a distance of 100 μm from each other) and the optical density of the extracellular matrix in the safranin O stain (conventional units). The first two indicators could be determined on reconstructed 3D images. The optic density of the cartilage for 3D reconstructed images was calculated as the equivalent of the optical density of the ECM by the formula *I* = 0.299R + 0.587G + 0.114B, where R, G and B are the average values of the color indicators in a selected area of cartilage. Quantitative data was processed using the Statistica 10.0 program (StatSoft Inc., USA) with the calculation of indicators adopted for the characterization of nonparametric samples in biomedical research: median [1st quarter, 3rd quartile]. To prove the reliability of the differences, we used the variance analysis using the nonparametric Friedman criterion for multiple groups (*p* < 0.05). The nonparametric Mann–Whitney test was used to analyze the differences between the two independent samples.

### Cryopreservation of biological objects

All models used in this experimental study underwent a similar process of flash-freezing, consequent embedding and secondary flash freezing. A cryogenic chamber, capable of maintaining low working temperatures was used for preparation of biological objects. The dimensions of the cryogenic chamber were 200 × 100 × 100 cm. No chemical treatment was used for preparation of biological objects prior to embedding. Liquid nitrogen at a temperature of − 196 °C was used for flash-freezing of biological material. All flash frozen biological material was maintained at a temperature below − 196 °C until further preparation was carried out. Flash-frozen and cryogenically stored biological objects underwent embedding with a cryogenic medium. The *Thermo* *Scientific* *Richard-Allan* *Scientific* *Neg-50* *Frozen* *Section* *Medium* was used for embedding of biological objects. Biological tissue was placed in the medium within the cryogenic chamber and flash-frozen as a compound and maintained at a working temperature of − 30 to − 35 °C.

### Cryogenic milling device

The structure of the cryogenic milling device developed for this procedure is available in Figs. 4, 5.^23^ The parameters required for proper functioning at cryogenic temperatures included separation of the cryogenic chamber from automated components of the device. Therefore two separate systems were incorporated into one: the cryogenic system for the interaction between the biological object and the mill, and the room-temperature system, which consisted of all other parts of the milling device. The specially designed milling device can be reconstructed according to the figures and must be cryogenically capable, maximum rotational force at 5000 rpm. The cryogenic milling device in our study weighed approximately 4000 kg, the high weight was mostly due to special anti-vibration additions to the structure. The step of sequenced milling was designed to provide as little as a 2–5 μm step between layers. This difference between layers is significant enough for microscopic histological evaluation and more than sufficient for macroscopic reconstruction. The default working step is 10 μm. With the given working parameters, constituted by the cryogenic chamber dimensions (200 × 100x100cm), the speed of complete milling of the object (at a rate of 19–24 s per surface) was approximately 48 h. Each mill passage is accompanied by temperature stabilization (cooling to working temperature). The milling device structure accounts for possible seismic, vibrational and resonance hindrance.

### Digitization

Photographic capturing of each consequent novel layer of the biological device was performed after each mill passage step. Depending on the step, the number of images produced varied from 2000 to 25,000 per object. Digitization was carried out by a Canon 5D camera, alternatively Canon 5DSR camera. The installation of the camera is operator dependent, but automated. Any digital camera can be used for digitization, as well as a microphotography camera (e.g. modified Canon digital LSR camera). Image capturing is performed from outside the cryogenic chamber though an access window providing clear visualization of the surface of the milled cryogenic model. The digital images are immediately transferred to a computer, where they are stored and used for virtual reconstruction. The camera placement is outside the cryogenic chamber. Apart from the camera, the digitization system includes lighting and stabilization features. Adaptation to new equipment is easily performed, as even a hand held device is capable of digitizing surface images, though produced image quality would be inconsistent. Overall, the entirety of image capturing is completely modifiable and depends on researcher preferences. Most commonly, the following settings were used: Camera Canon 5D with Canon EOS5D objective, with a pixel size 8 × 8 μm.

### Virtual reconstruction

After digitization each image is simultaneously transferred to the computer for storage and editing. For creating a three dimensional model from a series of layered images, we used Selena software, version 8.0. Selena stores each image in .bio format according to position in the model (consecutively). Any program allowing for modification, color management and three-dimensional rendering of stacked images can be applied for virtual reconstruction. The choice of software was constricted by funding limitations and can be changed according to researcher preference. Any software capable of working with an input of a series of sequenced images and producing a three-dimensional model is necessary for virtual reconstruction of the acquired data. This makes our method highly modifiable and accessible in different institutions, accommodating for different approaches and processing techniques. Generally, the software carries out the task of image alignment, outline matching, manual optimization, scaling, multidimensional rotation, removal of molding material (embedding medium), virtual slicing. The stacked images were acquired with nearly homogenous slice thickness. Selena software alternatives that were considered, but could not be used due to funding restriction were: Amira (available at https://www.thermofisher.com) and Imaris (available at https://imaris.oxinst.com), both offering the ability for virtual reconstruction of an image sequence.

Computerized processing of the acquired images is performed through software based on Modex computing core and has the ability to provide online 3D models with virtual slicing and sectioning in desired planes, constructing raster and wireframe models for source images of slices. The computational core implements algorithms of analytic geometry and vector algebra to find lines of intersection of the surface of the wireframe model with section planes. According to the raster model of the object, pixels are found that lie in a section, which in the general case is a plane in general position. The cross-sectional textures are constructed using the geometrical parameters of the cross-section plane and the color characteristics of the pixels found. Since the wireframe model of the object is a collection of faces defining the cross-section planes relative to the raster model, the general texture of the entire model is determined in the same way.

### Aspects of software processing

**Figure 14:**
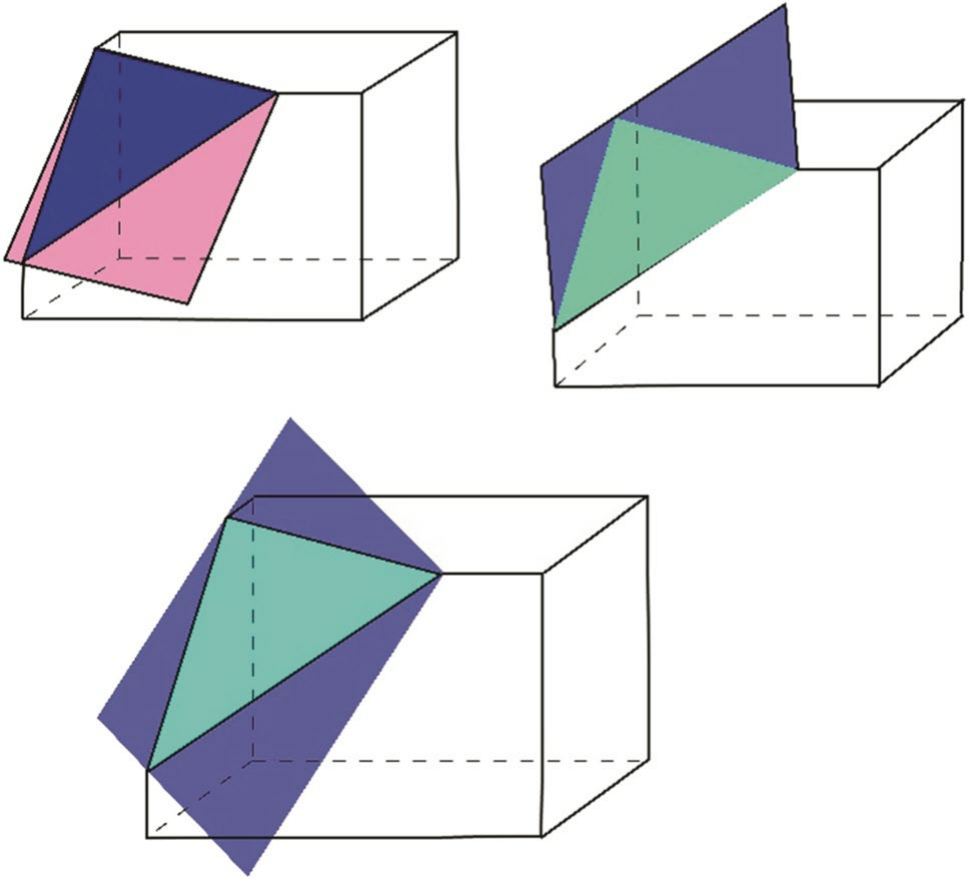
Examples of Θ-plane in triangular sections.

As a result of cyrogenic milling and surface image capturing, an image sequence is created with a certain step (Δz), with a resolution determined by sample size and operator modified camera settings. Further processing requires stacking of images and additional rendering, including creation of plane-based sectioning ( of the reconstructed 3D object. In order to create plane-based sectioning, we introduce a polygon plane (*Θ-plane)*, which can be altered and transformed according to research goals (Fig. 14).

After applying the RGB color system, each element of the mapping color table (*P*) is considered as a vector in three-dimensional space. Thereby, the vector field Ω^v^(*x*, *y*, *z*) = {*R*, *G*, *B*} within a region of the sample space converts it into a color space. To construct an image on the sample slice plane, the *Θ*-plane of this section must to be divided into pixels. Each pixel is associated with a color depending on its spatial position in a vector field Ω^v^. A set of sliced images is the initial data: *Z*_*k*_ = (*k *− 1) *Δz* , where the k-th image number (*k* = 1, …, *M*) is determined by the z-coordinate of the histological section. Consider a numbered set of raster images of size *P*_*x*_ × *P*_*y*_, where *P*_*x*_ and *P*_*y*_ are the numbers of pixels for the two corresponding sides. This initial set of images is designated “digital set of base slices”. To reconstruct an object in the form of a three-dimmensional model, a method for color interpolation in the space between each adjoining pair of images in the plains *Z*_*k*_ and *Z*_*k*+1_ is specified. The entire set of initial images is considered as a 3D matrix (*P* = (*i,j,k*)), whose elemens are individual pixels of images from the set. Their addressing is determined by the number of the *k*-th layer (image sequence number), the number of the *i*-th rowand the number of the *j*-th column in the matrix of pizels of the *k*-th photo. Discrete functions defined on a three-dimensional grid (*x*_*i*_*,* *y*_*j*_*,* *z*_*k*_) are used. The statement that the vector *ξ* is orthogonal to the normal vector to the plane described by the equation A*x* + B*y* + C*z* + D = 0 is equivalent tantamount to *Aξx* + *Cξz* = 0.

The vector *η* obeys the conditions of orthogonality to the vector *ξ* and the normal section plane. This means that its direction can be computed as the vector product of the vector *ξ* by the normal vector to the section plane:

$$\eta = \left[ {\xi , n} \right] = det\left( {\begin{array}{*{20}c} {e_{x} } & {e_{y} } & {e_{z} } \\ {\xi_{x} } & {\xi_{y} } & {\xi_{z} } \\ A & B & C \\ \end{array} } \right),$$

where *n* is the normal vector to the plane of the section, e_x_, e_y,_ e_z_ are the unit vectors along the positive directions of the x-, y- and z-axes respectively in the reference system associated with the sample. Taking into account that vector *ξ* and *η* have a unit length, the following equations can be drawn out:

$$\begin{aligned} & \sqrt {\xi_{x}^{2} + \xi_{z}^{2} } = 1, \\ & \sqrt {\eta_{x}^{2} + \eta_{y}^{2} + \eta_{z}^{2} } = 1. \\ \end{aligned}$$

From these equations we can calculate the coordinate values of vecotrs *ξ* and *η:*

$$\begin{aligned} & \xi = \left\{ {\frac{C}{{\surd \left( {A^{2} + C^{2} } \right)}}, 0, - \frac{A}{{A^{2} + C^{2} }} } \right\}, \\ & \eta = \left\{ { - \frac{AB}{L}, \frac{{A^{2} + C^{2} }}{L}, - \frac{BC}{L}} \right\}, \\ & L = \sqrt {A^{2} B^{2} + \left( {A^{2} + C^{2} } \right)^{2} + B^{2} C^{2} } . \\ \end{aligned}$$

In the reference system associated with the section plane we denote *p* and *q* the unit vectors along the direction of the abscissa and ordinate axes, respectively. The vector *p* is orthogonal to the unit vector directed along the applicate axis in the reference systome associated with the sample *p*_*z*_ = *0,* where *p*_*z*_ is the projection of the vector *p* onto the given axis. It is therefore required to add equations denotin the orthogonality of the vector *p* to the normal vector to the plane of the section, and the orthogonality of the vector *q* to both of them, as well as condition that the length of the vectors *p* and *q* is equal to one:

$$\begin{aligned} & Ap_{x} + Bp_{y} = 0, \\ & q = det\left( {\begin{array}{*{20}c} {e_{x} } & {e_{y} } & {e_{z} } \\ {\xi_{x} } & {\xi_{y} } & {\xi_{z} } \\ A & B & C \\ \end{array} } \right), \\ & \sqrt {p_{x}^{2} + p_{y}^{2} } = 1, \\ & \sqrt {q_{X}^{2} + q_{y}^{2} + q_{z}^{2} } = 1. \\ \end{aligned}$$

From these equations we can determine the values of the coordinates of the basis vectors in the reference system associated with the sample:

$$\begin{aligned} & p = \left\{ { - \frac{B}{{\sqrt {A^{2} + B^{2} } }}, \frac{A}{{\sqrt {A^{2} + B^{2} } }}, 0} \right\}, \\ & q = \left\{ { - \frac{AC}{M}, - \frac{BC}{M}, \frac{{A^{2} + B^{2} }}{M}} \right\}, \\ & M = \sqrt {A^{2} C^{2} + B^{2} C^{2} + \left( {A^{2} + B^{2} } \right)} . \\ \end{aligned}$$

These equations specify the direction of the basis vectors of such reference system, associated with the cross-section plane, for which the natural order of pixel bypass would guarantee the optimal number of calls to information recorded on the hard disk. As a result cross sectioned images can be obtained from an image stack.

**Figure 15:**
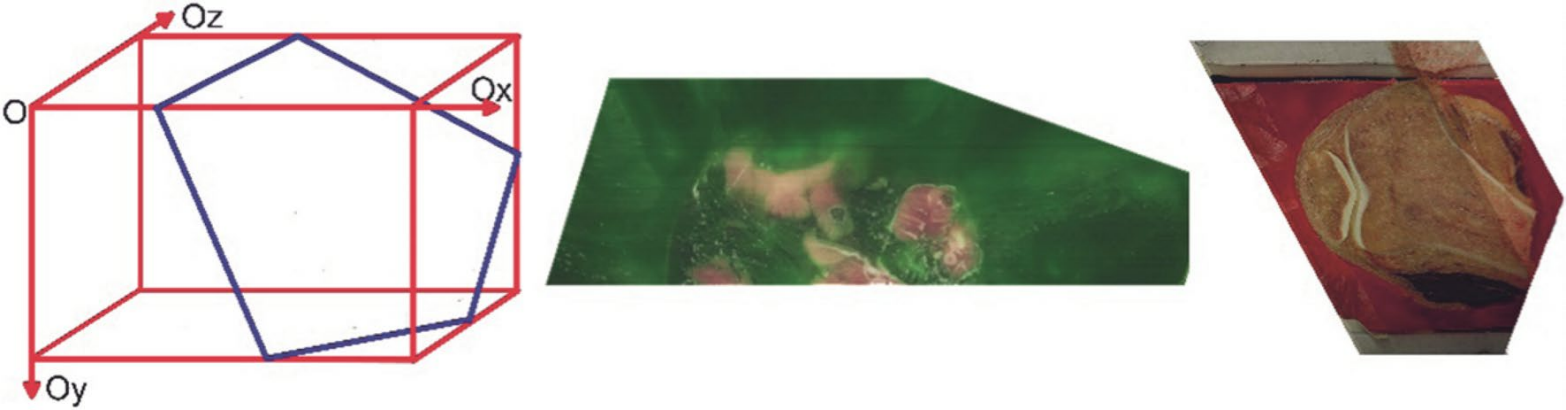
Example of the incorporated virtual slicing algorithm on a biological model.

The incorporated algorithm results in a manageable and virtually sliceable three-dimensional model (Fig. 15).

### Modeling software development

The software developed at out laboratory includes two main components: graphical user interface (GUI) program and modules for virtual slicing of reconstructed model. Microsoft Visual Studio 2012 comprising C# programming language with .NET framework 4.5 has been utilized as the software development environment. The programming interface was refined by API WPF. Data input is designated by a user-selected source (file containing image sequence). Further setting of geometric parameters of the sample are performed. Upon input, the program creates an object with specified dimensional characteristics (length, width and depth) and plane object (frontal face of the reconstructed object). The normal vector is co-directed with the OZ axis and applied to the origin—(0, 0, 0). The proposed method to construct vector 3D models of samples that are necessary for theoretical modeling of processes in highly heterogenous biological tissues can be applied using the given algorithm with any appropriate software.

### Application of acquired data

Upon researcher request, our method provides a series of real-life quality and texture images in any format, most commonly .TIFF, .JPG, .PNG. The image series can serve as basis for software layering and virtual reconstruction, animation, characterization, differentiation of tissue layers and other numerous applications. Image quality is modifiable, default camera settings produced 4,368 × 2,912 pixel images. Each image carries important topography-anatomical information, which is useful in modeling of surgical procedure, educational visualization and diagnostics. Educational and diagnostic application of cryogenic sequenced layering can be seen in the modeling of morphofunctional characteristics of the “joint-synovial fluid” system, a published PhD thesis (available at https://dlib.rsl.ru/viewer/01008706918#?page=1).

### Data assessment

Quality of tissue preservation was analyzed based on several factors: overall tissue integrity, degree of tissue damage, presence of low-temperature deformities (such as crystallization), RGB-profile. Subjective analysis of tissue integrity was performed. Computerized analysis of RGB-profile analysis was performed using open access “ImageJ” software and Statistica 10.0 software for analysis of results^24^.
